# A systematic review of assessment approaches to predict opioid misuse in people with cancer

**DOI:** 10.1007/s00520-022-06895-w

**Published:** 2022-02-15

**Authors:** Robyn Keall, Paul Keall, Carly Kiani, Tim Luckett, Richard McNeill, Melanie Lovell

**Affiliations:** 1 HammondCare, Sydney, Australia; 2 ACRF Image X Institute Sydney, University of Sydney, Sydney, Australia; 3 University of Western Australia, Perth, Australia; 4 Faculty of Health, University of Technology, Sydney, Australia; 5 Mary Potter Hospice, Wellington, New Zealand; 6 Department of Clinical Pharmacology, Christchurch Hospital, Christchurch, New Zealand

**Keywords:** Cancer, Opioid, Pain, Screening, Drug misuse

## Abstract

**Context:**

Cancer prevalence is increasing, with many patients requiring opioid analgesia. Clinicians need to ensure patients receive adequate pain relief. However, opioid misuse is widespread, and cancer patients are at risk.

**Objectives:**

This study aims (1) to identify screening approaches that have been used to assess and monitor risk of opioid misuse in patients with cancer; (2) to compare the prevalence of risk estimated by each of these screening approaches; and (3) to compare risk factors among demographic and clinical variables associated with a positive screen on each of the approaches.

**Methods:**

Medline, Cochrane Controlled Trial Register, PubMed, PsycINFO, and Embase databases were searched for articles reporting opioid misuse screening in cancer patients, along with handsearching the reference list of included articles. Bias was assessed using tools from the Joanna Briggs Suite.

**Results:**

Eighteen studies met the eligibility criteria, evaluating seven approaches: Urine Drug Test (UDT) (*n* = 8); the Screener and Opioid Assessment for Patients with Pain (SOAPP) and two variants, Revised and Short Form (*n* = 6); the Cut-down, Annoyed, Guilty, Eye-opener (CAGE) tool and one variant, Adapted to Include Drugs (*n* = 6); the Opioid Risk Tool (ORT) (*n* = 4); Prescription Monitoring Program (PMP) (*n* = 3); the Screen for Opioid-Associated Aberrant Behavior Risk (SOABR) (*n* = 1); and structured/specialist interviews (*n* = 1). Eight studies compared two or more approaches. The rates of risk of opioid misuse in the studied populations ranged from 6 to 65%, acknowledging that estimates are likely to have varied partly because of how specific to opioids the screening approaches were and whether a single or multi-step approach was used. UDT prompted by an intervention or observation of aberrant opioid behaviors (AOB) were conclusive of actual opioid misuse found to be 6.5–24%. Younger age, found in 8/10 studies; personal or family history of anxiety or other mental ill health, found in 6/8 studies; and history of illicit drug use, found in 4/6 studies, showed an increased risk of misuse.

**Conclusions:**

Younger age, personal or familial mental health history, and history of illicit drug use consistently showed an increased risk of opioid misuse. Clinical suspicion of opioid misuse may be raised by data from PMP or any of the standardized list of AOBs. Clinicians may use SOAPP-R, CAGE-AID, or ORT to screen for increased risk and may use UDT to confirm suspicion of opioid misuse or monitor adherence. More research into this important area is required.

**Significance of results:**

This systematic review summarized the literature on the use of opioid misuse risk approaches in people with cancer. The rates of reported risk range from 6 to 65%; however, true rate may be closer to 6.5–24%. Younger age, personal or familial mental health history, and history of illicit drug use consistently showed an increased risk of opioid misuse. Clinicians may choose from several approaches. Limited data are available on feasibility and patient experience.

PROSPERO registration number.

CRD42020163385.

## Introduction

### Cancer as a chronic disease

Cancer diagnoses worldwide are increasing, with over 18 million new cases and over 9 million deaths in 2018 [1]. Whilst continuing to be the second highest cause of death, thanks to new treatment options, the death rate has dropped by 24%, with 7 out of 10 people surviving for 5 years from diagnosis [2].

### Cancer pain

Pain affects 39% of people with potentially curable cancer, 55% of people whilst undergoing curative or palliative treatment, and 66% of those with advanced and or metastatic cancer [3]. Opioids are the pharmacological treatment of choice for cancer pain that is not responsive to simple analgesics [4].

### Opioid misuse

Opioid misuse or non-medical use is defined as use of a legally prescribed drug for euphoric rather than analgesic effect, using it in a manner or strength that has not been prescribed or using someone else’s prescribed drug (i.e., “diverting” it) [5].

### Opioid prescription and misuse

The extension of opioids for use in chronic non-cancer pain (CNCP) resulted in 168 million opioid prescriptions in the USA in 2018 [6] and a 15-fold increase in opioid prescriptions dispensed in Australia between 1992 and 2012 [7]. The rate of prescription of opioids is increasing in older people with cancer [8]. As legal prescriptions have increased, so have hospitalizations and deaths related to opioids and/or illicit drugs, with 46,802 deaths attributed to prescription opioids alone in the USA in 2018 [9] and approximately 1,000 in 2018 in Australia [10]. It is estimated that 4% of the USA population over 12 years old and 16 million people worldwide have misused prescription pain medication. Each person who misuses opioids costs USD$9,000–16,000 more in healthcare costs compared to non-misusers [11, 12]. Opioid misuse is a major public health problem created by legitimate prescribers and providers and exacerbated by psychosocial and economic issues [13]. Misuse of prescribed and unprescribed opioids is now one of the major international health crises of the twenty first century [14].

### Opioid misuse risk versus opioid misuse

In this systematic review, we report on both “opioid misuse,” which is demonstrated through evidence, and “opioid misuse risk,” which can be assessed using a patient questionnaire (directly answered or retrospectively applied from medical documentation) and results in a score which identifies patients as being at high or low risk of opioid misuse.

### Opioid prescription misuse and cancer patients

A recent systematic review of substance use disorder in cancer patients found median rates of 18% previous or current opioid misuse and 25.5% alcohol misuse [15]. This higher prevalence of alcohol misuse in cancer patients may reflect a causal pathway, given the metabolites of ethanol are a carcinogen [16]. Risk of opioid misuse can increase with chronicity of treatment [17]. Although the risk of opioid misuse has historically been thought to be low when opioids are prescribed for cancer, there is increasing concern that the risks are, in fact, significant and under-recognized. Concern has led to the development of guidelines for clinicians advising use of harm minimization strategies [4]. However, in a recent survey of palliative care clinicians, most reported having patients with opioid misuse risk and lacked the confidence to adequately manage or mitigate the associated risk, with less than a quarter using any screening tools or strategies [18]. General practitioners may be less concerned about the risk of opioid misuse in cancer patients compared to CNCP, potentially posing a risk due to the under-recognition of this problem [19].

Mitigation strategies are most efficient when employed proportionately to the risk of opioid misuse. Accordingly, this requires a means of screening patients for opioid misuse. Guidance on how to screen patients for opioid misuse should be evidence-based. However, most guidance around screening for clinicians is based on evidence from prescribing for CNCP, and it is unclear how this applies to prescribing for cancer pain.

We conducted a systematic review to address this evidence gap. The aims of this review were (1) to identify screening approaches that have been used to assess and monitor risk of opioid misuse in patients with cancer; (2) to compare the prevalence of risk estimated by each of these screening approaches; and (3) to compare risk factors among demographic and clinical variables associated with a positive screen on each of the approaches.

## Methods

The systematic review was registered with PROSPERO (registration number CRD42020163385) and was performed in accordance with PRISMA guidelines [20].

### Eligibility criteria

In population, intervention, comparator, outcomes, and study (PICOS) terms, inclusion criteria were adolescent and/or adults with a diagnosis of cancer and prescribed or considered for prescription of opioids for pain (P); with risk mitigation interventions or strategies to predict or prevent opioid misuse (I); with any or no comparator (C); prevalence of risk for opioid misuse (O); and randomized controlled trials, cohort studies, case–control studies, case series, and audits (S). Articles were included if published in English (or English-translated) in a peer-reviewed journal.

### Information sources

Between March and May 2020, a systematic search was undertaken of the following databases: Medline, Cochrane Controlled Trial Register, PubMed, PsycINFO, and Embase. Reference lists of included articles were searched by hand and eligible articles added.

### Search

The search terms used were “cancer pain,” “neoplasm,” “cancer patient*.mp” AND “opioid*,” “opiate.mp” AND “harm minimization,” “risk mitigation,” “brief risk questionnaire,” “urine drug screen,” “SOAPP-R,” “ORT,” “COMM,” “pain medicine questionnaire,” “abuse deterrent opioid formulation.”

### Study selection

Abstracts were retrieved and duplicates removed. Two independent reviewers (R. K. and C. K.) reviewed all the abstracts, and agreement was made on eligible articles to be retrieved in full. Differences were discussed and with a third and fourth party (R. M. and M. L.), and consensus articles were retrieved in full by R. K. and C. K.

### Data extraction

Data items were extracted by three reviewers (R. M., C. K., and R. K.) including setting, sample characteristics, intervention, research design, comparator, and prevalence of misuse and associated risk factors.

### Risk of bias in individual studies

The reviewers assessed bias using the Joanna Briggs Suite of tools [21].

### Synthesis

Because of heterogeneity between samples and screening approaches, synthesis used a narrative approach [22]. Three authors (R. M, C. K., and R. K.) synthesized the findings of the individual tools in both a tabulated form for numerical comparison and in discussion.

## Results

**Fig. 1:**
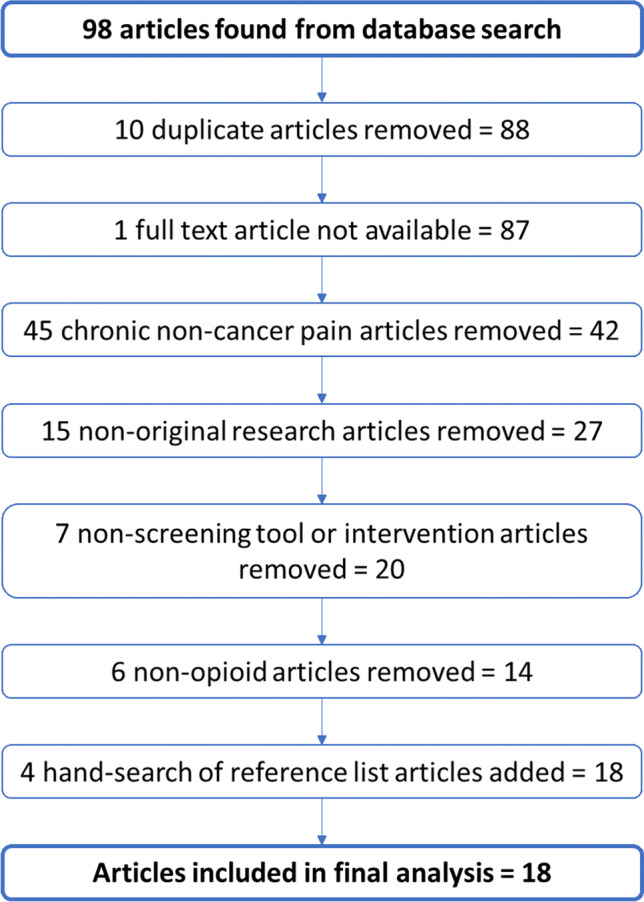
Flow diagram of the article selection process

The searches yielded 98 articles, of which 14 met the criteria and were included, along with four more identified by hand-searches (*N* = 18). See Fig. 1 for detail.

### Study characteristics

Of the 18 studies, 14 were case series (8 retrospective), two retrospective cohort studies, one a retrospective case–control study, and one, a case report. There were no randomized controlled trials. All studies were conducted in the USA.

### Screening approaches in patients with cancer

The studies evaluated seven assessment approaches to assess for opioid misuse in patients with cancer: Urine Drug Test (UDT) (*n* = 8); the Screener and Opioid Assessment for Patients with Pain (SOAPP) and two variants, Revised and Short Form (*n* = 6); the Cut-down, Annoyed, Guilty and Eye-opener (CAGE) tool and one variant, Adapted to Include Drugs (*n* = 6); the Opioid Risk Tool (ORT) (*n* = 4); Prescription Monitoring Program (PMP) (*n* = 3); the Screen for Opioid-Associated Aberrant Behavior Risk (SOABR) (*n* = 1); structured/specialist interviews (*n* = 1); and compared one or more tools (*n* = 8). Thienprayoon et al. (2017) [23] and Anghelescu et al. *(*2013) [24] both mention the use of a patient interview but did not describe this in any detail, therefore could not be analyzed. The screening approaches that have been used to assess and monitor risk of opioid misuse in patients with cancer are described briefly below.

### Screening interventions used in patients with cancer

#### Urine Drug Test (UDT)

UDT can be conducted at the bedside or in the laboratory. Point-of-care UDT can identify the presence or absence of a class of drug and is affordable with a high sensitivity but low specificity [25]. More reliable but also costlier and time-consuming is gas chromatography mass spectrometry. These tests can provide information about specific drugs and their metabolites [26]. False positives can be observed in people with conditions associated with high lactic acidosis, ingestion of poppy seeds, and some medicines, whilst people wishing to avoid detection may refuse, over-hydrate, or add substances to their specimen [26, 27].

Although evidence is limited for UDT to assess risk and monitor opioid treatment, it has been recommended in both the cancer [4] and non-cancer pain population [28]. There are no studies comparing UDT against no UDT as a risk mitigation strategy [29].

#### The Screener and Opioid Assessment for Patients with Pain (SOAPP)

The original SOAPP tool consists of 14 questions about behaviors associated with opioid misuse, each scored on a Likert scale from zero (“never”) to four (“very often”). A total score of seven or more indicated high risk of opioid misuse. The SOAPP was shown to have adequate sensitivity and specificity [30]. Two revised forms of the SOAPP tool have been developed, both retaining the zero to four Likert scale. Screener and Opioid Assessment for Patients with Pain-Revised (SOAPP-R) was created to improve the sensitivity and specificity of the tool by increasing the number of questions to 24, with highly significant reliability and predictability [31], whereas the Screener and Opioid Assessment for Patients with Pain-Short form (SOAPP-SF) improved clinical feasibility by reducing the questions to five but also increased the risk of a false positive response by a third [31, 32]. A total score of 18 or more was considered positive for SOAPP-R, and a score of four or more was positive for SOAPP-SF.

#### The Cut-Down, Annoyed, Guilty, Eye-Opener (CAGE)

The CAGE asks four questions about behaviors associated with alcohol misuse. Answering yes to two or more of the four questions is considered positive. The CAGE is an effective screening tool for alcoholism in many settings with varying success [33–37], with sensitivity between 0.71 and 0.84 and specificity of 0.9–0.95 [38]. Whilst there is one highly cited study to show daily alcohol use being a risk factor of prescription drug abuse, there is limited evidence elsewhere to support this claim [39, 40]. This limited evidence for use of CAGE to identify opioid misuse risk has led to the development of the Cut-Down, Annoyed, Guilty, Eye-Opener-Adapted to Include Drugs (CAGE-AID), first validated in 1995 [41] and found to be more sensitive but less specific for substance misuse than the CAGE [42]. The CAGE-AID has expanded the 4 CAGE questions to include drug use in the questions, i.e., “Have you ever you felt the need to Cut down on your drinking or drug use?” Answering yes to one or more questions is considered positive and further evaluation should be undertaken. The questions refer to a person’s lifetime usage and drug use refers to both illicit and misuse of legal drugs.

#### The Opioid Risk Tool (ORT)

The ORT consists of 10 items, resulting in a score from 0 to 24. Patients are assessed as low (0–3), moderate (4–7 s), or high risk (> 8). Categories are personal or family history of substance abuse, age, history of pre-adolescent sexual abuse, and a history of specific psychiatric disorders. Although validated in chronic non-cancer pain, a recent systematic review concluded that the ORT did not predict aberrant drug behavior in chronic pain due to the low quality of the included studies [43]; another paper found inconsistency in results with sensitivity ranging from 0.20 to 0.99 and specificity from 0.16 to 0.88 [29]. The ORT has not been validated in the cancer population.

#### Prescription Monitoring Program (PMP)

PMPs are databases which record details, from a variety of sources, about prescriptions including the prescriber, the medication, and the recipient. PMPs can alert prescribers in real time or be searched to obtain information. PMPs are not universally available. In a recent systematic review of the effectiveness of PMPs, the authors found limited evidence of reducing opioid prescribing, dispensing, and multiple prescribers [44].

#### Screen for Opioid-Associated Aberrant Behavior Risk (SOABR)

The SOABR tool was intended for use in adolescents and young adults with cancer [24]. It assesses the presence or absence of six risk factors, including substance misuse, mental health diagnoses, and a history of sexual abuse. As this tool has not been validated to date, interpretation is less established than for other tools.

Table 1 summarizes the included studies’ designs, settings, sample characteristics, screening intervention(s), findings regarding the prevalence of opioid misuse, and comments. The studies are grouped by screening approach. Rates of increased opioid misuse risk in the studied populations ranged from 6 to 65%.

**Table 1:** Study characteristics

Study	Study design	Setting	Sample, cancer type and size	Sample mean age (years/SD)	Screening/approach	Prevalence of opioid misuse
Arthur et al. (2020) [45]	Retrospective cohort study	Supportive care clinic	Cancer- not detailed type *n* = 300	Random UDT IQR = 60 (48–63) Targeted UDT IQR = 53 (40–61)	Random UDT or UDT based on CS & CAGE-AID	Abnormal UDT for presence of unprescribed opioid = 35/300 (12%) CAGE-AID positive = number not defined CAGE-AID positive and all abnormal UDT = OR 2.29 (1.10–4.77) (*p* = 0.03)
Koyyalagunta et al. (2018) [46]	Retrospective cohort study	Pain clinic	Mixed cancer, 27% with no evidence of disease *n* = 167	49 ± 12 (compliant) 46 ± 12 (non-compliant)	SOAPP-SF & UDT as part of pre-prescribing practice	Abnormal UDT for the presence of unprescribed opioid = 36/167 (22%) SOAPP-SF HR = 108 (65%) SOAPP-SF HR score ≥ 4 had higher rates of non-adherence to prescribed opioids (*p* = 0.0403)
Rauenzahn et al. (2017) [47]	Retrospective case series	Supportive care clinic	Cancer, 43% no evidence of active disease *n* = 82	56.2 ± 12.7 (normal UDT) 48.2 ± 9.9 (abnormal UDT)	UDT based on CS	Abnormal UDT for presence of unprescribed opioid = 20/82 (24%)
Yasin et al. (2019) [48]	Retrospective case series	Palliative care clinic	Mixed cancers *n* = 69	Not defined	SOAPP-R, ± UDT, ± AOB recorded	SOAPP-R HR = 27 (39.1%), SOAPP-R had a sensitivity of 0.75, specificity of 0.80, PPV = 0.67, NPV = 0.86 Abnormal UDT not further defined = 15 (22%) AOBs = 24 (29%)
Thienprayoon et al. (2017)[23]	Case series	Palliative care inpatient team and ambulatory clinic	Pediatric and young adults with solid and hematological cancers, and non-cancer disorders *n* = 62	Range 4–28	UDT & PMP as part of opioid risk bundle	HR of opioid misuse stratification = 21(34%), MR of opioid misuse. stratification = 16 (27%), abnormal UDT = number not defined
Childers et al. (2015) [49]	Retrospective case series	Cancer pain and supportive care clinic	37% CNCP, mixed cancers *n* = 57 UDT *n* = 27	54 ± 13	SOAPP-SF & CAGE ± UDT based on CS	SOAPP HR = 26 (46%), CAGE positive = 5 (15%), UDT abnormal for presence of unprescribed opioids = 4/27 (15%)
Barclay et al. (2014) [50]	Retrospective case series	Palliative Care clinic	Cancer- not detailed type *n* = 114 UDT *n* = 46	53, SD not available	UDT & ORT	ORT HR = 24 (21%), ORT MR = 25 (22%) UDT abnormal for presence of unprescribed opioids = 3/46 (6.5%) All abnormal UDT was lower in ORT LR than MR/HR (*p* = 0.0005)
Arthur et al. (2016) [51]	Case reports	Palliative care clinic	Inactive colon cancer, active HCC *n* = 2	“Middle-aged” and “elderly”	CS, PMP, UDT & CAGE-AID	CAGE-AID positive = 1/2, abnormal UDT for presence of unprescribed opioids = 1/2
Yennurajalingam et al. (2018) [52]	Case series	Supportive care clinic	Mixed cancers *n* = 729	Median 61, SD not available, IQR 53–69	SOAPP, CAGE-AID	SOAPP HR = 143(19.6%), CAGE- AID positive = 73 (10.5%) SOAPP HR and CAGE-AID positive = (*p* = 0.001)
Koyyalagunta et al. (2013) [53]	Retrospective case series	Pain clinic/palliative care	Mixed cancers, 13% without evidence of disease, *n* = 522	56 ± 14 (LR) 50 ± 14(HR)	SOAPP-SF	SOAPP-SF HR = 149(29%) SOAPP-SF score ≥ 4 predicted higher opioid doses at presentation (but not at subsequent visits)
Reyes-Gibby et al. (2016) [54]	Case series	Emergency Department	Mixed cancers, solid and blood borne *n* = 209	54.2 ± 13.1	SOAPP-R, PMP	SOAPP-R HR = 71(34%), PMP data of number of annual prescriptions high versus low risk on SOAPP-R = 17.8 vs 12.6; *p* = 0.023
Anghelescu et al. (2013) [24]	Case series	Specialist pain service- pediatric anesthetics	Mixed cancers and sickle cell disease *n* = 38	Median 19	Diagnostic interview ± SOAPP-R	SOAPP-R HR = 3/13(39.5%) Diagnostic interview HR = 15/38 (32%) 3/7 high risk patients developed AOBs compared to 0/6 standard risk patients
Dev et al. (2011) [56]	Retrospective case control	Supportive care clinic	Solid and blood borne cancers *n* = 598	Mean 58.6 (CAGE positive) Average 61.3 (CAGE negative)	CAGE	CAGE positive = 100(17%) CAGE positive more likely to have history of IDU (*p* < 0.001)
Kim et al. (2016) [55]	Retrospective case series	Supportive care clinic	Advanced cancers *n* = 300	60 ± 13	CAGE Smoking status IDU statues	CAGE positive = 49 (16%) History of IDU = 39(13%) Current smokers = 33 (11%) Current smokers + CAGE positive + Hx IDU (*p* < 0.001)
Garcia et al. (2017) [58]	Case series	Palliative care clinic and Gynecologic oncology clinic	Gynecological *n* = 118	Median 57, SD not available	ORT	ORT MR/HR = 15 (13%)
Ma et al. (2014) [57]	Retrospective case series	Palliative care clinic in a cancer center	Mixed cancers, 6% sickle cell disease *n* = 114	53 ± 13	ORT & PMP	ORT HR = 21 (25%), ORT MR = 28 (18%)
Teulings & Broglio, (2020) [65]	Case series	Cancer clinic	Head and neck cancers *n* = 40	Mean 64 Range 45–81	ORT	ORT HR = 4(10%) ORT MR = 8(20%) History of substance misuse = 8(20%)
Ehrentraut et al. (2014) [60]	Retrospective case series	Hematology/oncology outpatients	Mixed cancers, sickle cell disease (1%) *N* = 94	16.3 ± 2.82	SOABR, AOBs	AOB observed = 11.7% SOABR did not predict AOBs *p* = 0.165)

Key: *AOBs*, aberrant opioid behaviors; *CAGE*, the Cut-down, Annoyed, Guilty and Eye-opener; *CAGE-AID*, the Cut-down, Annoyed, Guilty and Eye-opener-Adapted to Include Drugs; *CS*, clinical suspicion; *HR*, High Risk; *IDU*, illicit drug use; *IQR*, inter-quartile range; *LR*, low risk; *Mod.*, moderate quality; *NPV*, negative predictive value; *ORT*, Opioid Risk Tool; *OR*, odds ratio; *PPM*, Prescription Monitoring Program; *PPV*, positive predictive value; *SOABR*, Screen for Opioid-Associated Aberrant Behavior Risk; *SOAPP*, Screener and Opioid Assessment for Patients with Pain; *SOAPP-R*, Screener and Opioid Assessment for Patients with Pain-Revised; *SOAPP-SF*, Screener and Opioid Assessment for Patients with Pain-Short Form; *UDT*, Urine Drug Test

### Urinary Drug Test

Of the included studies, eight used UDT to screen for or identify opioid misuse based on clinical suspicion raised by observation of aberrant opioid behaviors (AOBs) or positive result on screening tool [23, 24, 45–50]. Four studies used targeted UDT [47, 49–51], two studies used UDT for all patients [23, 48], one reviewed a screening tool against previously recorded but motivation unknown UDT [46], and one study compared targeted UDT to random UDT [45]. In the reviewed studies, UDTs were more commonly abnormal due to the presence of illicit or non-prescribed opioids rather than the absence of a prescribed drug. For this review, only data for inclusion of unprescribed opioids was considered as evidence of opioid misuse; this was defined in 5 studies [45–47, 49, 50] and 1 case study [51]; we consider that although the absence of a prescribed opioid may point to diversion, it may reflect patient choice. Therefore, the actual rates of opioid misuse shown by UDT in the five studies ranged from 6.5 to 24%, aggregate of 98/652 (15%).

Of the included studies, UDT was used for primary screening as part of usual practice [23, 45, 46] and/or secondary screening after risk identified by (i) another tool ORT [50], CAGE [47], CAGE-Adapted to Include Drugs (CAGE-AID) [51], or (ii) clinical suspicion based on history of drug misuse and/or aberrant opioid behaviors and/or high symptom expression on validated tool [45, 47, 49–51].

Clinical suspicion was the reason for the majority of the UDT. Koyyalagunta et al. (2018) screened both opioid-naïve and opioid-familiar patients referred by other clinicians as routine practice before prescribing opioids [46]. Arthur et al. (2020) explored the strength of clinical suspicion over random selection in their cohort study, finding an almost twofold increase in abnormal UDTs [45].

### Screener and Opioid Assessment for Patients with Pain (SOAPP)

Seven included studies used one of the forms of the SOAPP tool as a screening intervention for patients with cancer [24, 46, 48, 49, 52–54]. Most were case series apart from one retrospective cohort study.

Yennurajalingam et al. (2018) studied 729 adults in the context of comparing the CAGE-AID to the validated SOAPP. One hundred forty-three out of 729 (19.6%) participants were found to have high risk per SOAPP [52].

Three studies used the SOAPP-SF [46, 49, 53]. Childers et al. (2015) conducted a prospective study in a palliative care clinic using SOAPP-SF and found that 46% of patients had a positive score. Over 90% of patients had a cancer diagnosis, but just over a quarter had non-cancer pain. Those with non-cancer pain were more likely to also complete a UDT and account for 75% of the abnormal UDT results. Though the abnormal UDTs were further classified to show unexpected opioids present, there was no statistically significant findings to understand those patient characteristics. The authors did not discuss any correlation between the two screening tools [49].

SOAPP-SF was also used in two retrospective chart reviews by the same lead author. Koyyalagunta et al. (2013) reported the findings from over 500 patients at a cancer pain clinic in which the SOAPP-SF is integrated into usual practice and 13% had a history of illicit drug use (IDU) [53]. SOAPP-SF indicated 29% of patients were high risk. Of their second case series of 167 patients who were also subjected to UDT, 65% (97/167) were abnormal [46]. Abnormal UDT was further defined to those with unexpected opioid inclusions 36/167 (22%) and all abnormal UDTs correlated with positive SOAPP-SF, in particular the question “how often have you used illegal drugs in the last 5 years?” The authors posit that this direct question alone may be adequate to stratify risk in this population [46].

Two case series used SOAPP-R. Anghelescu et al. (2013) presented a small case series of young adults from a specialist pain center. Thirteen patients completed the SOAPP-R, and this was combined with a psychologist assessment. Seven of these 13 patients were classified as high risk using both the psychologist assessment and SOAPP-R [24]. Yasin et al. (2019) describe their findings of 69 adults with cancer who completed the SOAPP-R; some underwent UDT based on clinical suspicion, and some had documentation of AOBs. The authors describe AOBs and found the SOAPP-R to have a sensitivity of 0.75 and specificity of 0.80 for opioid misuse [48].

A larger, prospective series was presented by Reyes-Gibby et al. (2016), who used the SOAPP-R to screen 209 patients with cancer already receiving opioids and attending the emergency department. One hundred ninety-eight out of 209 had their PMP data reviewed. Thirty-three percent of patients were classified as high risk using the SOAPP-R, and those with a positive SOAPP-R had received more prescriptions as recorded on the state-wide PMP (*p* = 0.023). 14.8% of patients had a history of IDU, and 3.8% currently used illicit drugs. This study also concluded that the SOAPP-R with its 24 questions was feasible [54].

### CAGE and CAGE-AID

Mixed results were found in the included studies, from no correlation between CAGE positive and abnormal UDT [49] to statistically significant correlation with smoking history [55] and/or illicit drug use through history taking [55, 56].

Arthur et al.’s (2020) cohort study used CAGE-AID and clinical suspicion to conduct UDT, finding a CAGE-AID positive correlated with abnormal UDT [45]. However, it is not clear whether CAGE-AID results alone were enough to prompt a UDT. Yennurajalingam et al. (2018) compared CAGE-AID to SOAPP and found a sensitivity of 43.9% and specificity of 90.9% and positive predictive value of 53.2% [52].

### Opioid Risk Tool (ORT)

Four studies used the ORT in patients with cancer. Two studies, Barclay et al. (2014) and Ma et al. (2014), retrospectively reviewed the charts of cancer patients and found an average of 21.5% for both moderate and high risk of opioid misuse [50, 57]. Barclay et al. (2014) had results of 46/114 participants who had also had a UDT. Abnormal UDT were present in 62.5% of those stratified as medium to high risk but also 7% of the 57% of total participants assessed as low risk. After corroboration of the ORT by the UDT, Barclay et al. (2014) found family history of alcohol misuse and personal history of IDU were most predictive of opioid misuse [50].

The remaining two studies were a prospective case series and a pilot feasibility study in narrower groups of cancers, gynecological and head and neck, respectively. Garcia et al. (2017) surveyed women with gynecological cancers with the lowest prevalence of all the included studies. Only 7% were classified as moderate and 6% as high risk, those with cervical cancer more likely than most to be high risk [58]. Whilst Teulings et al. (2020), studied the ORT in those with head and neck cancers, a population were associated with higher-than-average alcohol and smoking rates though not necessarily opioid misuse, and 20% were classified as moderate risk and 10% high [58]. The strongest predictors of scoring high risk on ORT were depression, family or personal alcohol misuse, younger age, and smoking, though this may reflect the subgroup of head and neck cancer patients that are associated with tobacco and alcohol use [59].

### Prescription Monitoring Program (PMP)

Reyes-Gibbs et al. (2016) used their state PMP to compare with the results of the SOAPP-R, finding statistically significant correlation with high SOAPP-R scores and higher than expected number of prescriptions and prescribers [54]. Arthur et al. (2016) used PMP data and UDT to confirm suspicion of AOBs [51].

### Screen for Opioid-Associated Aberrant Behavior Risk (SOABR)

Anghelescu et al. (2013) used correlation between AOB and both the presence of any risk factor, and the number of risk factors present to create the SOABR [24].

The SOABR tool has only been used in a single study to date, a retrospective case series of 94 patients with an oncological or hematological malignancy. Ehrentraut et al. (2014) report 10 of the 11 patients who demonstrated AOB scored at least one point using the SOABR tool [60].

### Clinical suspicion

Clinical suspicion of opioid misuse was mentioned in several studies. What raises clinical suspicion was not fully explained in all of the studies but may include issues such as AOB, including requests for lost prescriptions, missing appointments, requests for escalations, telephone calls for frequent refills [46, 47, 51] patients without active cancer or with non-cancer pain [49], and history of drug misuse [47, 50]. Yasin et al. (2019) propose a separate list of five AOBs, but of note, one of these includes a history of drug misuse which could be considered a risk factor rather than a current AOB [48]. Neither list has been definitively validated, although the 17-point list has a clear derivation reported based on literature of adults with chronic non-cancer pain. See Table 2 for a list of AOB based on the literature [24, 48, 60].

**Table 2:** Aberrant opioid behaviors compiled from included articles

Category	Behavior example
Observable behaviors	Not keeping appointments, requesting scripts from multiple prescribers, requests for scripts via phone, claiming lost or stolen scripts, pill count irregularities, asking for drug by street name, distress if drug unavailable, resistance to change plan
Medication non-compliance	Self-increasing dose or frequency, self-medicating for non-analgesic effect including euphoria, anxiety, and sleep
Interpersonal behaviors	Hoarding drugs, concerns by caregivers and/or family, decreased level of functioning, requiring drug to be able to function
Illegal behaviors	Illicit drug use, stealing, selling, or forging prescriptions, sourcing drugs illegally

### Correlations between screening tools and other factors in identifying opioid misuse risk

Three factors have the capacity to provide definitive evidence of opioid misuse: AOB, PMP, and UDT. Three studies correlated a screening tool with AOBs. In one small study, SOAPP-R combined with a specialist psychology review was associated with a higher rate of AOBs, but no statistical analysis was conducted [24]. In another study, SOAPP-R had a specificity of 0.75 and sensitivity of 0.80 when compared to a combination of AOB and UDT [48]. A study comparing SOABR and AOBs found no statistically significant correlation [24]. PMP data was correlated with the SOAPP-R tool in a single study; higher SOAPP-R scores were associated with more frequent opioid prescriptions [54]. UDT has been correlated with CAGE-AID [45], SOAPP-SF [46], and the ORT [50]. There was significant heterogeneity in these studies in abnormal UDT results, including the presence of unprescribed opioid, the absence of prescribed opioid, and the presence of illicit non-opioid drugs. Two further studies measured clinical outcomes. One measured AOBs but did not correlate these with the CAGE and SOAPP-SF results, perhaps due to the small sample size [49]. Another found that high SOAPP-SF scores correlated with high opioid doses at initial presentation to their pain clinic, but the correlation was not maintained at subsequent visits and is not an established marker of opioid misuse [53].

The articles identified varied in the weight that certain factors convey to increase clinical suspicion. Younger age, found to be significant in 8/10 studies, personal or family history of anxiety or other mental ill health, found to be significant in 6/8 studies, and history of illicit drug use, found to be significant in 4/6 studies, showed an increased risk of misuse. See Table 3 for factors identified as increasing risk.

**Table 3:** Demographic and clinical factors identified by included articles as associated with risk of opioid misuse

Risk factor identified to increase risk of opioid misuse	Approaches used from included articles showing statistical significance *p* < .05	Approaches used from included articles not showing statistical significance *p* < 0.05
Younger age	UDT [45–47], SOAPP-SF [46, 49, 52, 53], CAGE [56]	ORT [58, 57]
Personal or family history of anxiety or other mental ill health	UDT [45–47, 52], SOAPP-SF [53], SOAPP-R [54]	SOABR [60], ORT [57]
History of IDU	ORT [50], CAGE [56, 55], SOAPP-R [54]	ORT[57], UDT [46]
Gender (male)[46]	CAGE [56], UDT [45], ORT [50]	SOABR [60], UDT [46], SOAPP-SF [53], ORT [57]
Active use nicotine	CAGE [56, 55]	ORT [58, 57]
History of alcohol use	CAGE [55]	UDT[47], ORT [57]
Cancer type or treatment status	CAGE [56]	ORT[58], SOAPP-SF 29,[53], UDT [46], ORT [57]
Concurrent use of two or more opioids	SOABR [60]	
Single, divorced, or never married		ORT [57]
History of pre-adolescent sexual abuse (women)		ORT [57], SOABR [60]
Family history of alcohol use		ORT [50], SOABR [60], ORT [57]
MEDD	UDT [45]	SOAPP-SF [53], UDT [47], CAGE [56]
Expression of pain/symptoms	SOAPP-SF [52, 53]	SOAPP-SF [49], CAGE[46, 49, 58], ORT[57], UDT [46]
Race	UDT[47]	CAGE [56]
Socioeconomic status	SOAPP-SF [53]	UDT [46]

Key: *CAGE*, the Cut-down, Annoyed, Guilty and Eye-opener; *IDU*, illicit drug use; *MEDD*, morphine equivalent daily dose; *ORT*, Opioid Risk Tool; *SOABR*, Screen for Opioid-Associated Aberrant Behavior Risk; *SOAPP-R*, Screener and Opioid Assessment for Patients with Pain-Revised; *SOAPP-SF*, Screener and Opioid Assessment for Patients with Pain-Short Form; *UDT*, urine drug test

## Discussion

This systematic review of assessment approaches to predict opioid misuse in people with cancer summarizes the existing research on screening for opioid misuse in patients with cancer. Seven approaches have been identified. Four are clinical tools, SOAPP, ORT, SOABR, and CAGE (and their variants), used to identify risk factors; the remainder are dedicated specialist assessments or interviews and PMP and UDT which can confirm misuse. A high percentage (average 45%) of the illicit drug detected in the abnormal UDT was cannabis; in many cases, it was the only aberration, and in some geographical regions, cannabis may be decriminalized or legally prescribed. Rates of opioid misuse risk from the included studies ranged from 6 to 65% with confirmed opioid misuse through UDT (presence of non-prescribed opioid only was 6.5–24% [24, 45, 46, 48, 50, 53, 60]. SOAPP-R, ORT, and CAGE-AID were more reliable than CAGE, SOAPP, SOABR, and SOAPP-SF in identifying increased risk as confirmed by AOB, PMP, or UDT. Younger age, personal or familial mental health history, and history of illicit drug use consistently showed an increased risk of or actual opioid misuse. Clinical suspicion may be raised by any of the list of aberrant opioid behaviors drawn from the literature. UDT, AOBs, and PMP data may be used to confirm clinical suspicion and/or monitor adherence to treatment.

The only two studies to report the rate of AOBs in their whole population both report rates of 10–15%, although these studies were both small and limited to a specific population of adolescents and young adults with specialist cancer pain [24, 60]. Two later studies from the same center, identified during the peer review process and so not included in this review, provide early evidence of a similar rate in adults with cancer. One used universal screening for AOBs and found a prevalence of 19% [61], although a single AOB may not reflect true opioid misuse. The second used random UDT and found 15% had an abnormality when cannabis was excluded, although other illicit drugs were included [62]. In both studies, SOAPP and CAGE-AID were statistically correlated with the presence of an AOB, but positive and negative predicative values were not reported. The two studies in adolescents and both adult studies were conducted in single centers and all in the USA [24, 60–62]. Coupling AOB rates with abnormal UDT rates from the included studies suggest the rates of opioid misuse in the cancer patient population to be closer to 15%; however, this rate comes from a small number of studies. It would be useful to confirm these findings in other centers and countries. Data from screening questionnaires suggest risk factors are prevalent in the cancer population, and between 6 and 65% of patients are classified as high risk. This large variation suggests screening questionnaires likely overestimate the true risk of opioid misuse in this population. A 2015 systematic review of opioid misuse risk in the chronic non-cancer pain population also found wide ranges of results from < 1 to 81% of increased risk [63].

The rates of opioid misuse identified by targeted UDT were high in all studies. The rates were similar whether UDT was ordered based on clinical suspicion alone or using a dedicated screening tool. There is insufficient evidence to support the routine use of screening interventions other than UDT. There is weak evidence to support the universal use of UDT to identify opioid misuse. However, UDT will only identify patients with established misuse and allow mitigation of harm to themselves or others rather than identifying patients at risk to prevent harm. The absence of prescribed drug raises concerns for diversion of drug: the bulk of people who take opioids not prescribed for them have been obtained from a friend or relative at no cost. These are generally entry-level misusers, whilst those that continue to misuse over a sustained period will likely source their opioids through drug dealer or physicians [64]. Screening for drug misuse and/or mental health in the home, as reported in one study, may be helpful in stratifying risk [23].

Several research gaps in today’s evidence have emerged from this review. Included studies were conducted in the USA alone. It is unclear whether other countries have similar rates of opioid risk. Future research should focus on correlating the results of screening interventions such as UDT and AOBs, with established diagnoses of opioid misuse. Feasibility studies are required to support the implementation of routine screening for opioid misuse from patient, clinician, and healthcare system perspectives. The psychosocial impact of both screening and subsequent management strategies on patients and clinicians is crucial to understanding the holistic impact of screening and risk mitigation strategies. More work is needed to understand whether, and how, identification of actual opioid misuse or assessed risk alters patient management.

There is more research into opioid misuse risk in the chronic non-cancer pain setting than cancer pain setting with existing practice guidelines [4]. For example, the current opioid misuse measure (COMM) tool is recommended to screen for opioid misuse in patients already receiving opioid therapy; however, there were no studies of the COMM in the cancer population identified by this review. There is no research to understand the psychosocial effects of screening and management of opioid misuse in cancer patients.

This systematic review had several limitations including the narrow search criteria to include only studies with active cancer diagnoses published in a peer-reviewed English language journal. The heterogeneity of the studies made comparative analysis challenging including detailing the reliability of some of the tools.

## Conclusions

This review found that the risk of opioid misuse varies between 6 and 65% depending on the tool used and the population screened, with rates of documented opioid misuse closer to 15%. Younger age, personal or familial mental health history, and history of illicit drug use consistently showed an increased risk of opioid misuse. Clinicians may choose to use SOAPP-R, ORT, or CAGE-AID to screen for increased risk of opioid misuse. Clinical suspicion of opioid misuse may be raised by data from PMPs if they are available or any of the standardized list of AOBs. Clinicians may use UDT to confirm suspicion of opioid misuse or monitor adherence, but UDT fails to identify those at risk. There is no research to understand the psychosocial effects of screening and management of opioid misuse. There remains an urgent need for further research in this area given the increasing rates of opioid prescription*.*

## Data Availability

Data are available, and method of accessing the same is transparent.
